# Swiss QUality of life and healthcare impact Assessment in a Real-world Erenumab treated migraine population (SQUARE study): interim results

**DOI:** 10.1186/s10194-022-01515-8

**Published:** 2022-11-18

**Authors:** Andreas R. Gantenbein, Reto Agosti, Christian P. Kamm, Gunther Landmann, Niklaus Meier, Gabriele Susanne Merki-Feld, Jens A. Petersen, Heiko Pohl, Philippe Ryvlin, Christoph J. Schankin, Dragana Viceic, Chiara Zecca, Elisabeth Schäfer, Ina Meyer, Michael E. Arzt

**Affiliations:** 1 Department of Neurology and Neurorehabilitation, ZURZACH Care, Bad Zurzach, Switzerland; 2 Kopfwehzentrum Hirslanden, Zurich, Switzerland; 3 Department of Neurology, Inselspital, University Hospital Bern and University of Bern, Bern, Switzerland; 4 Neurocentre, Luzerner Kantonsspital, Lucerne, Switzerland; 5 Center for Pain Medicine, Nottwil, Switzerland; 6 Department of Neurology, Spital Thun, Thun, Switzerland; 7 Department of Reproductive Endocrinology, University Hospital Zurich, Zurich, Switzerland; 8 Neurozentrum Bern, Bern, Switzerland; 9 Department of Neurology, University Hospital Zurich, Zurich, Switzerland; 10 Department of Clinical Neurosciences, CHUV, Lausanne, Switzerland; 11 Private Practice, Sion, Switzerland; 12 Neurocenter of Southern Switzerland, Ente Ospedaliero Cantonale (EOC), Lugano, Switzerland; 13 Faculty of Biomedical Sciences, Università Della Svizzera Italiana, Lugano, Switzerland; 14 Novartis Pharma Schweiz AG, Rotkreuz, Switzerland

**Keywords:** Erenumab, Migraine, Real-world evidence, Quality of life, Healthcare impact, Switzerland

## Abstract

**Background:**

The fully human monoclonal antibody erenumab, which targets the calcitonin gene-related peptide (CGRP) receptor, was licensed in Switzerland in July 2018 for the prophylactic treatment of migraine.

To complement findings from the pivotal program, this observational study was designed to collect and evaluate clinical data on the impact of erenumab on several endpoints, such as quality of life, migraine-related impairment and treatment satisfaction in a real-world setting.

**Methods:**

An interim analysis was conducted after all patients completed 6 months of erenumab treatment. Patients kept a headache diary and completed questionnaires at follow up visits. The overall study duration comprises 24 months.

**Results:**

In total, 172 adults with chronic or episodic migraine from 19 different sites across Switzerland were enrolled to receive erenumab every 4 weeks. At baseline, patients had 16.6 ± 7.2 monthly migraine days (MMD) and 11.6 ± 7.0 acute migraine-specific medication days per month. After 6 months, erenumab treatment reduced Headache Impact Test (HIT-6™) scores by 7.7 ± 8.4 (*p* < 0.001), the modified Migraine Disability Assessment (mMIDAS) by 14.1 ± 17.8 (*p* < 0.001), MMD by 7.6 ± 7.0 (*p* < 0.001) and acute migraine-specific medication days per month by 6.6 ± 5.4 (*p* < 0.001). Erenumab also reduced the impact of migraine on social and family life, as evidenced by a reduction of Impact of Migraine on Partners and Adolescent Children (IMPAC) scores by 6.1 ± 6.7 (*p* < 0.001). Patients reported a mean effectiveness of 67.1, convenience of 82.4 and global satisfaction of 72.4 in the Treatment Satisfaction Questionnaire for Medication (TSQM-9). In total, 99 adverse events (AE) and 12 serious adverse events (SAE) were observed in 62 and 11 patients, respectively. All SAE were regarded as not related to the study medication.

**Conclusions:**

Overall quality of life improved and treatment satisfaction was rated high with erenumab treatment in real-world clinical practice. In addition, the reported impact of migraine on spouses and children of patients was reduced.

**Trial registration:**

BASEC ID 2018–02,375 in the Register of All Projects in Switzerland (RAPS).

**Graphical Abstract:**

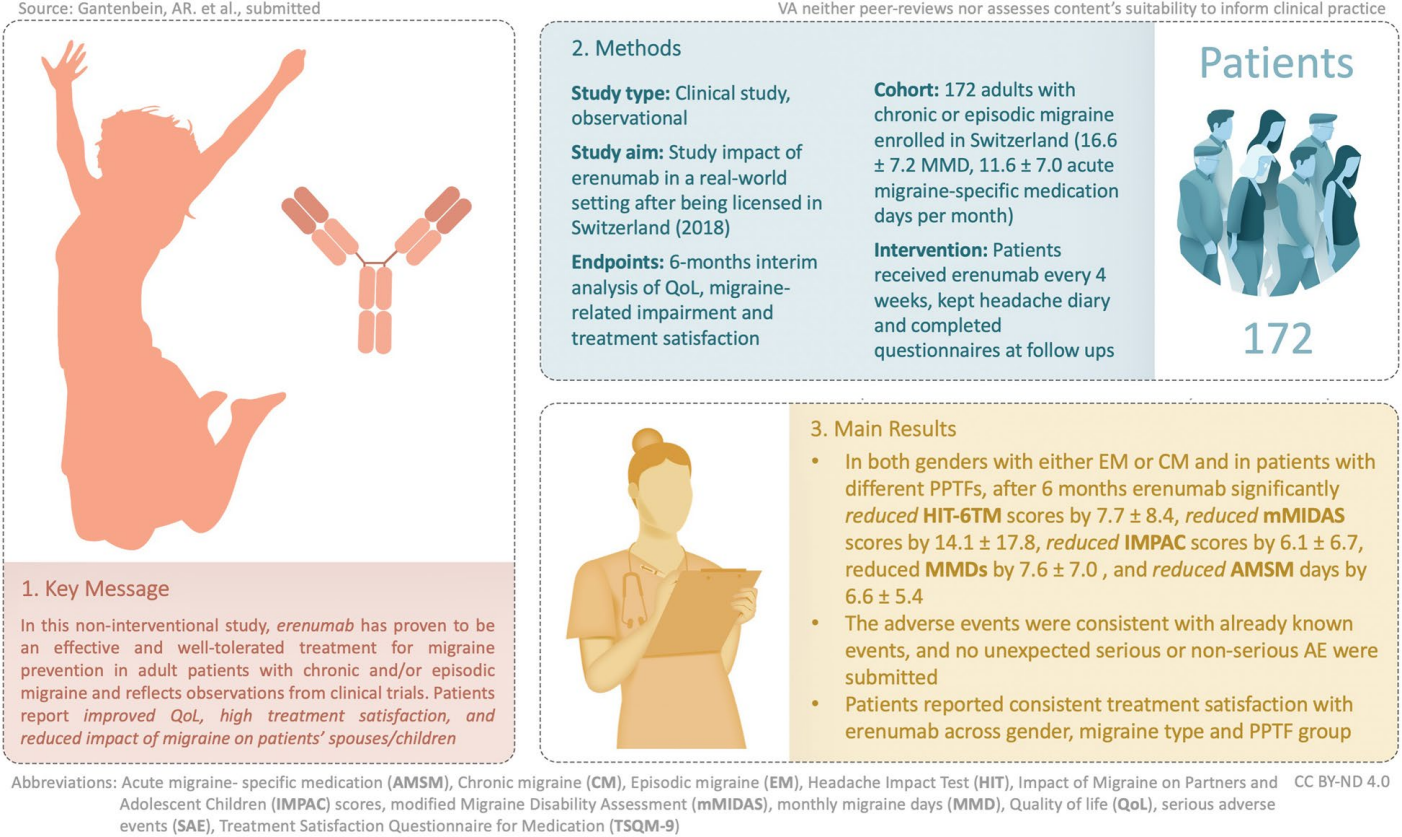

**Supplementary Information:**

The online version contains supplementary material available at 10.1186/s10194-022-01515-8.

## Introduction

Migraine, as the most common disabling neurologic disorder, is complex and long-lasting, with attacks varying in frequency and character [1, 2]. Migraine headaches are often accompanied by other symptoms, such as nausea, vomiting and hypersensitivity to different stimuli, compromising mobility and physical functioning, and limiting the participation in everyday activities [3]. Impairing the quality of life, migraine can result in substantial personal, economic and societal burden [4–7]. Patients with frequent migraine attacks are usually treated with a variety of preventive drugs. Many of these therapies are not approved for the preventive treatment of migraine because of insufficient or limited evidence [8]. Common drugs used for migraine prophylaxis include beta-blockers and other anti-hypertensives, anti-convulsive medication, anti-depressants, natural substances, hormones and others. Although these prophylactic medications can reduce headache frequency, duration, and severity, most have been repurposed from other indications and have not been designed to target the underlying pathophysiology of migraine [9]. In addition, adherence to these prophylactic therapies is generally poor, with many patients discontinuing due to safety, tolerability, and/or efficacy issues [10].

Erenumab, a fully human monoclonal antibody targeting the calcitonin gene-related peptide (CGRP) receptor, is a specific prophylactic treatment for adult patients with migraine to reduce the number of monthly migraine days (MMD) [11]. Global confirmatory studies have characterized the safety and efficacy of erenumab in patients with episodic (EM) and chronic migraine (CM) [12–14]. Remarkably, prophylactic treatment with 70 mg or 140 mg erenumab showed a consistent and statistically significant reduction of MMD, acute medication days, as well as significant improvement of quality of life versus placebo. The incidence of adverse events, serious adverse events, and discontinuation rate due to adverse events was comparable to placebo in these studies. Based on these data, erenumab received marketing authorization in Switzerland for the prevention of migraine in adults in July 2018.

Real-world data evaluating the effect of erenumab on multiple dimensions of quality of life in a setting of routine medical care are limited. The non-interventional SQUARE study (Swiss QUality of life and healthcare impact Assessment in a Real-world Erenumab treated migraine population) aims to address this gap by collecting data on the impact of erenumab treatment on patient-reported quality of life and migraine-related disability, as well as treatment satisfaction and persistence in a real-world setting. In order to more adequately describe the spectrum of constraints caused by migraine, the impact of migraine on partners and adolescent children of patients was also assessed.

In line with the primary outcome of this study (HIT-6™ at month 6 vs. baseline), this interim analysis at 6 months represents the first and primary communication of the SQUARE data. These results therefore represent real-world evidence from one of the first countries worldwide where erenumab was approved.

## Methods

This multicenter and non-interventional cohort study was designed to investigate the effects of erenumab on quality of life, migraine-related impairment and treatment satisfaction in a real-world setting.

The overall study duration is 24 months with a primary analysis 6 months after the end of patient recruitment.

Both migraine care specialist centers and general neurologists in all geographical regions of Switzerland were included in order to obtain a representative sample of the whole migraine treatment landscape.

**Fig. 1:**
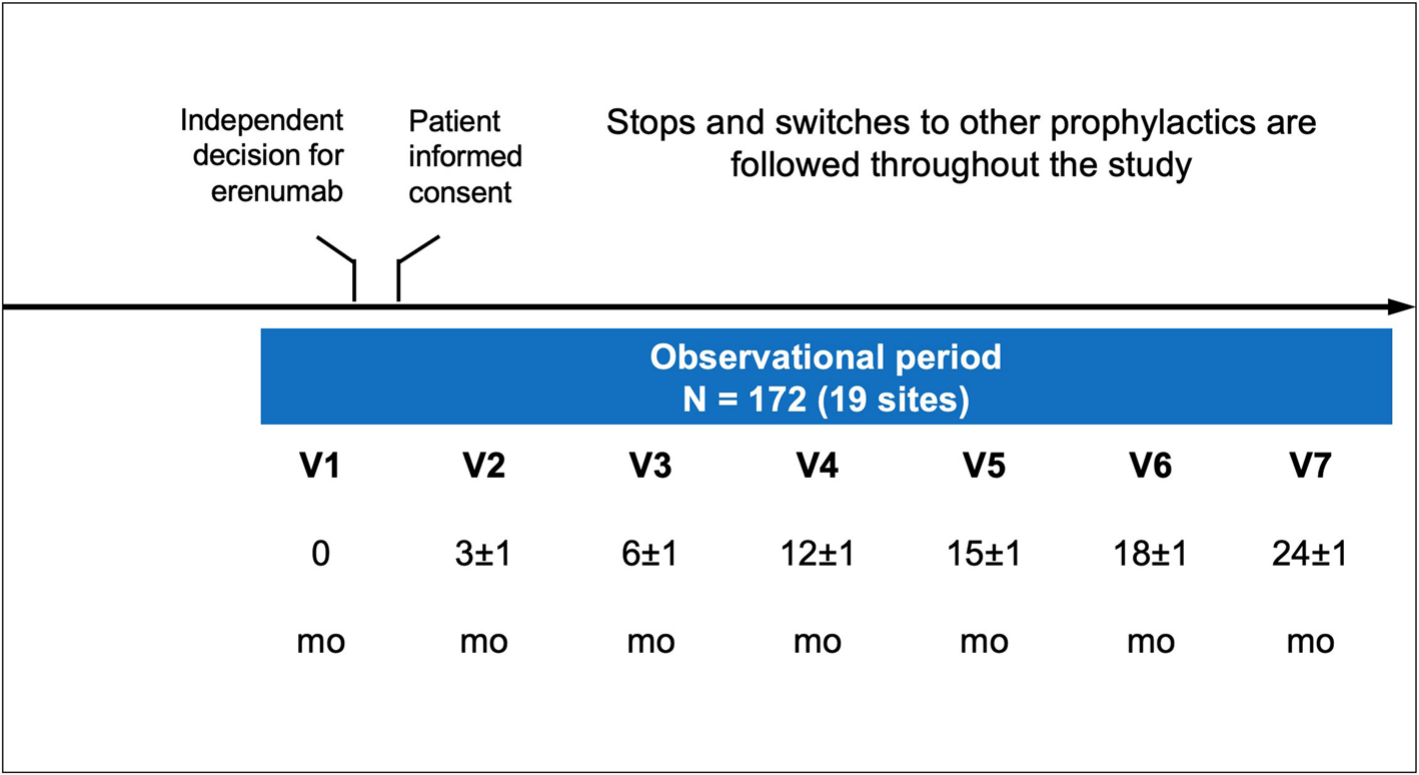
Study design and visit schedule. mo: month; V: visit

To comply with the non-interventional nature of this study, the visit schedule reflected recommendations only, with an acceptable window of ± 1 month for each assessment time point (Fig. 1).

Data were taken from medical charts, completed patient-reported outcome (PRO) questionnaires, and patient diaries.

Prospective data were collected in form of the following PRO questionnaires:

The Headache Impact Test (HIT-6™), to assess the severity and impact of migraine and associated impairment in everyday life during 4 weeks [15]. The higher the score (possible values ranging from 36 to 78), the greater the impact of the patient’s migraine is on daily life. A modified version of the Migraine Disability Assessment (mMIDAS) was used to assess headache-related disability. A recall of 30 days was chosen instead of 90 days as defined in the original MIDAS [16] to avoid overlap of MIDAS-scores due to the flexible visit windows. The mMIDAS classifies patients into four grades (ranging from grade I to IV with increasing disability). The social and especially family impact of migraine was measured by the Impact of Migraine on Partners and Adolescent Children (IMPAC) questionnaire [17]. IMPAC classifies patients into four grades (ranging from grade I to IV with increasing severity). By TSQM-9, the Treatment Satisfaction Questionnaire for Medication [18], the patient’s satisfaction with the medication was evaluated at month 6 (score ranging from 0 to 100 with increasing satisfaction). Additional patient-related data were collected from the patient’s medical chart prospectively during routine visits (e.g., concomitant medication, migraine days, intensity, acute medication, adverse events, health care utilization). Retrospectively collected data included demographics, medical history, and prior prophylactic treatment failures (PPTF). MMD, intensity of migraine, and acute medication days were collected from migraine diaries starting three months before initiation of erenumab treatment.

All adverse events (AE) – including serious adverse events (SAEs) and safety endpoints – were collected and recorded in the study database, irrespective of causal association. Subsequent prophylactic therapies are also understood as investigated drugs of this study because they are part of the investigational aim.

All treatment decisions were fully independent of the participation in this study.

Over the course of the study, designated staff entered collected parameters into an electronic Case Report Form (eCRF), using a fully validated, web-based software solution for capture of patient data (via the application Studymate^©^).

### Patients and setting

Patients participating in this study were recruited with the following inclusion criteria: adults with a diagnosis of migraine according to the International Classification of Headache Disorders (ICHD-3), signed informed consent, decision prior to enrolment that the patient will start erenumab treatment in alignment with the Swiss label (erenumab is indicated for prophylaxis of migraine in adults) [19]. Patients were included before or with the first injection and were able to record migraine attacks in a diary during the course of the study as well as PRO questionnaires.

Patients were excluded if they used investigational drugs during the study or within 3 months before enrolment or within 5 half-lives of investigational drug before enrolment or until the expected pharmacodynamic effect had returned to baseline, whichever was longer. Patients with prior treatment with erenumab or any anti-CGRP pathway therapy were also excluded.

### Statistical analysis

A sample size of 77 patients was calculated based on the observed variability of HIT-6™ in the BECOME [20] and STRIVE [12] studies. For within-group changes of HIT-6™, a minimum important difference of 3.7 has been reported in the literature [21]. Considering an expected drop-out rate of 60%, a total sample size of 193 patients was calculated.

Due to the observational character of this study, primarily descriptive methods were used.

All statistical test results were assessed at a significance level of *p* ≤ 0.05.

No data imputations were committed.

## Results

### Patient characteristics at baseline

A total of 172 patients at 19 sites were enrolled. Patient enrolment was stopped earlier than planned due to the COVID-19 pandemic. However, the achieved number of patients was sufficient for the statistical analysis due to lower drop-out rates than anticipated. The mean age of the study population was 44.2 ± 13.9 years. The majority of patients were women (*n* = 146; 84.9%). Patients had 16.6 ± 7.2 MMD and 11.6 ± 7.0 acute migraine-specific medication days per month at baseline. Episodic migraine was diagnosed in 92 patients (53.8%) and chronic migraine in 79 patients (46.2%) (Table 1).

**Table 1:** Demographics and baseline characteristics, working status of study population, migraine type and history

**Characteristics**	*N* = 172	
**Age**, mean (SD)	44.2 (13.9)	
**Sex**, n (%)		
Women	146 (84.9)	
**Working status**, n (%)		
Part-time employed	61 (35.5)	
Full-time employed	54 (31.4)	
Retired	14 (8.1)	
Sick leave/disability insurance	11 (6.4)	
In education/military service/civilian service	5 (2.9)	
**Monthly migraine days**, mean (SD)	16.6 (7.2)	
**Monthly acute medication days**, mean (SD)	11.6 (7.0)	
**Migraine history of patients initiating erenumab**, mean (SD)	Male	Female
Years with headache	33.2 (21.9)	27.3 (14.1)
Years since diagnosis	14.7 (14.4)	19.2 (14.7)
**Type of migraine according to ICHD-3**, n (%)		
Episodic migraine (EM)	92 (53.8)	
Low frequency EM (LFEM), 4–7 MMD	6 (3.5)	
High frequency EM (HFEM), 8–14 MMD	86 (50.3)	
Chronic migraine (CM)	79 (46.2)	

A combination of different migraine types was possible

*Abbreviations*: *CM* Chronic migraine, *EM* Episodic migraine, *HFEM* High frequency episodic migraine, *ICHD-3* International Classification of Headache Disorders, *LFEM* Low frequency episodic migraine, *MMD* Monthly migraine days, *SD* Standard deviation

On average, patients had 4.0 ± 1.9 prior prophylactic treatment failures (PPTF) before being started on erenumab (mostly, beta-blockers or topiramate). The vast majority of patients had two or more PPTFs (Table 2). Medications and substance classes of the most common PPTFs are listed in the Supplementary Information (Table S1).

**Table 2:** Prior prophylactic treatment Failures by count and percentage

**PPTF** Count, n (%)	*N* = 171
1 PPTF	5 (2.9)
2 PPTF	32 (18.7)
3 PPTF	42 (24.6)
4 PPTF	38 (22.2)
5 PPTF	26 (15.2)
≥ 6 PPTF	28 (16.4)

*Abbreviations*: *PPTF* Prior Prophylactic Treatment Failure

The average HIT-6™ score at baseline was 65.9 ± 4.9 points. Of all patients, 58.9% had severe disability equivalent to mMIDAS grade IV. An IMPAC grade of III was reported by 54.3% and a grade of IV by 32.7% of patients, indicating severe or very severe impact on their partners and adolescent children.

### Six-months results

#### Headache Impact Test-6 (HIT-6™)

**Fig. 2:**
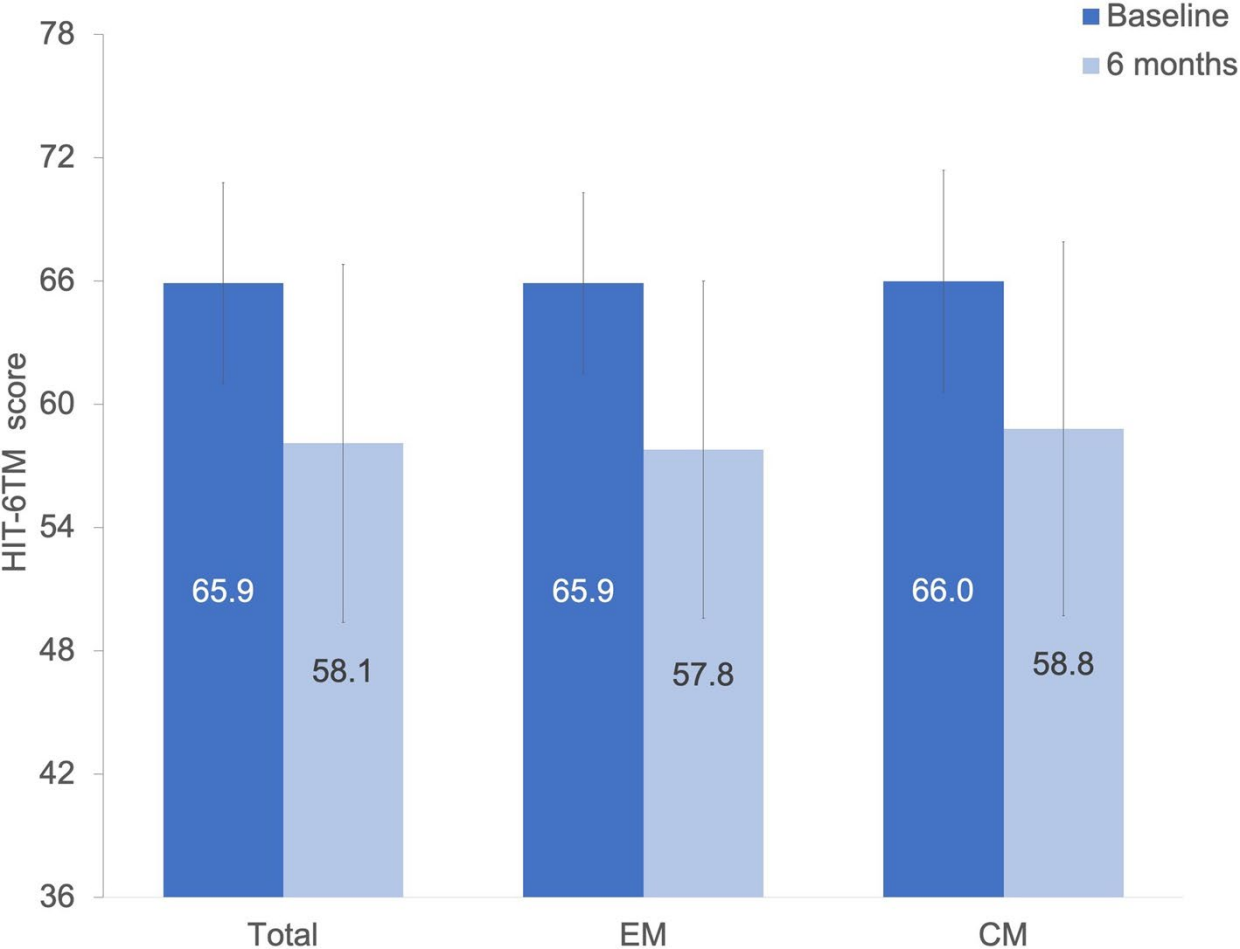
HIT-6™ reduction after 6 months per migraine type (*N* = 159). The full range of HIT-6™ possible scores is shown, with 36 being the lowest-possible score and 78 the highest-possible score. Abbreviations: EM: episodic migraine; CM: chronic migraine; HIT-6.™: Headache Impact Test-6

The reduction of the HIT-6™ score was 7.7 ± 8.4 compared to baseline after 6 months (*p* < 0.001) in the overall cohort of 159 patients. Separate analysis for 86 EM and 73 CM patients showed reductions of 8.2 ± 8.7 and 7.1 ± 8.1 after 6 months, respectively (Fig. 2).

#### Modified migraine disability assessment (mMIDAS)

**Fig. 3:**
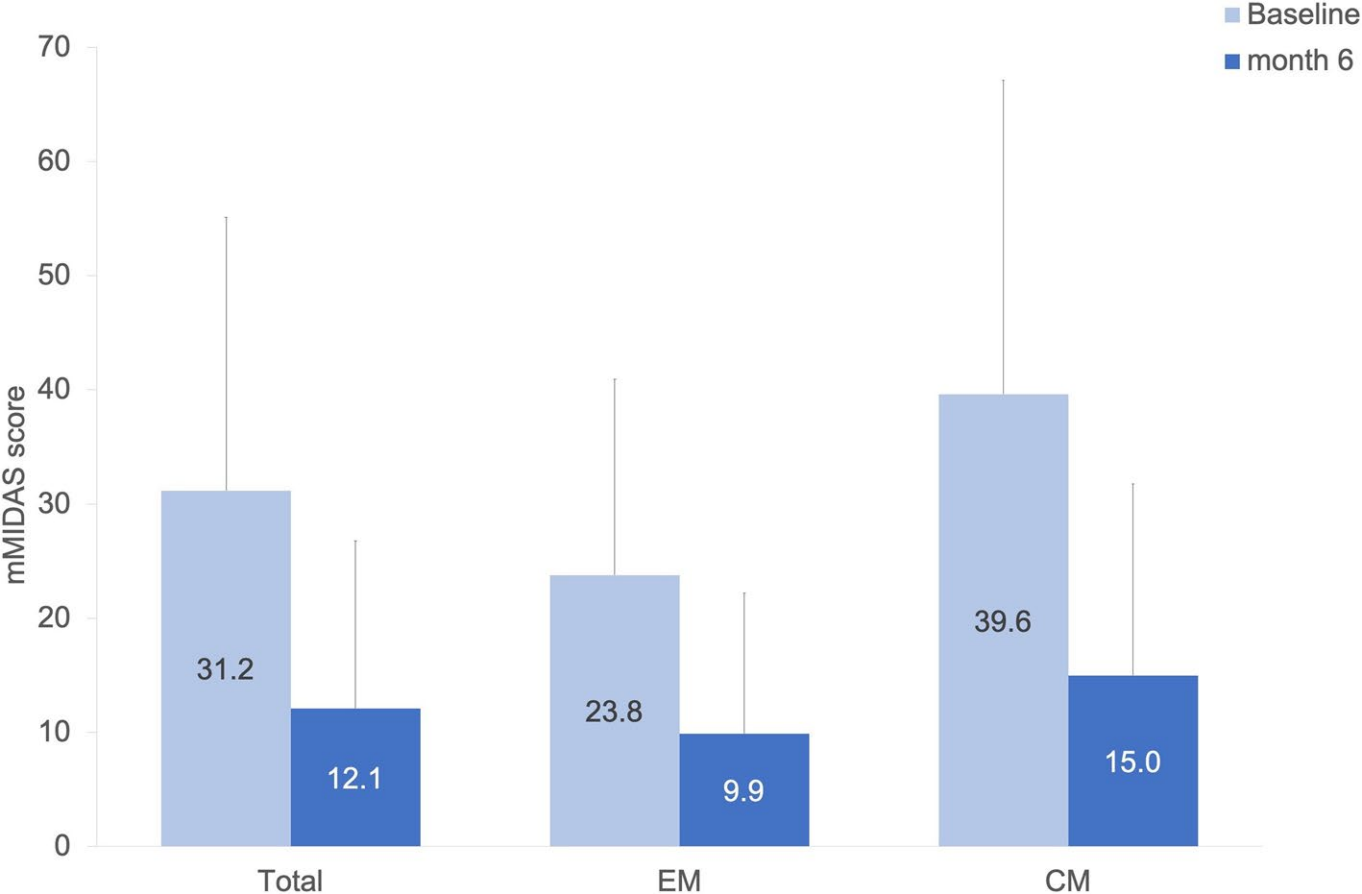
Mean mMIDAS score at baseline and after 6 months per migraine type (*N* = 151). Abbreviations: EM: episodic migraine; CM: chronic migraine; mMIDAS: modified migraine disability assessment

Overall mMIDAS decreased significantly from baseline to month 6 (*p* < 0.001) (Fig. 3). The mean reduction for the 82 EM patients in mMIDAS who had documented values at both time points was 14.1 ± 17.8 compared to baseline (23.8 ± 17.1), and for the 69 CM patients 23.6 ± 25.1 compared to baseline (39.6 ± 27.5), with *p* < 0.001, respectively.

#### Impact of Migraine on Partners and Adolescent Children (IMPAC Score)

**Fig. 4:**
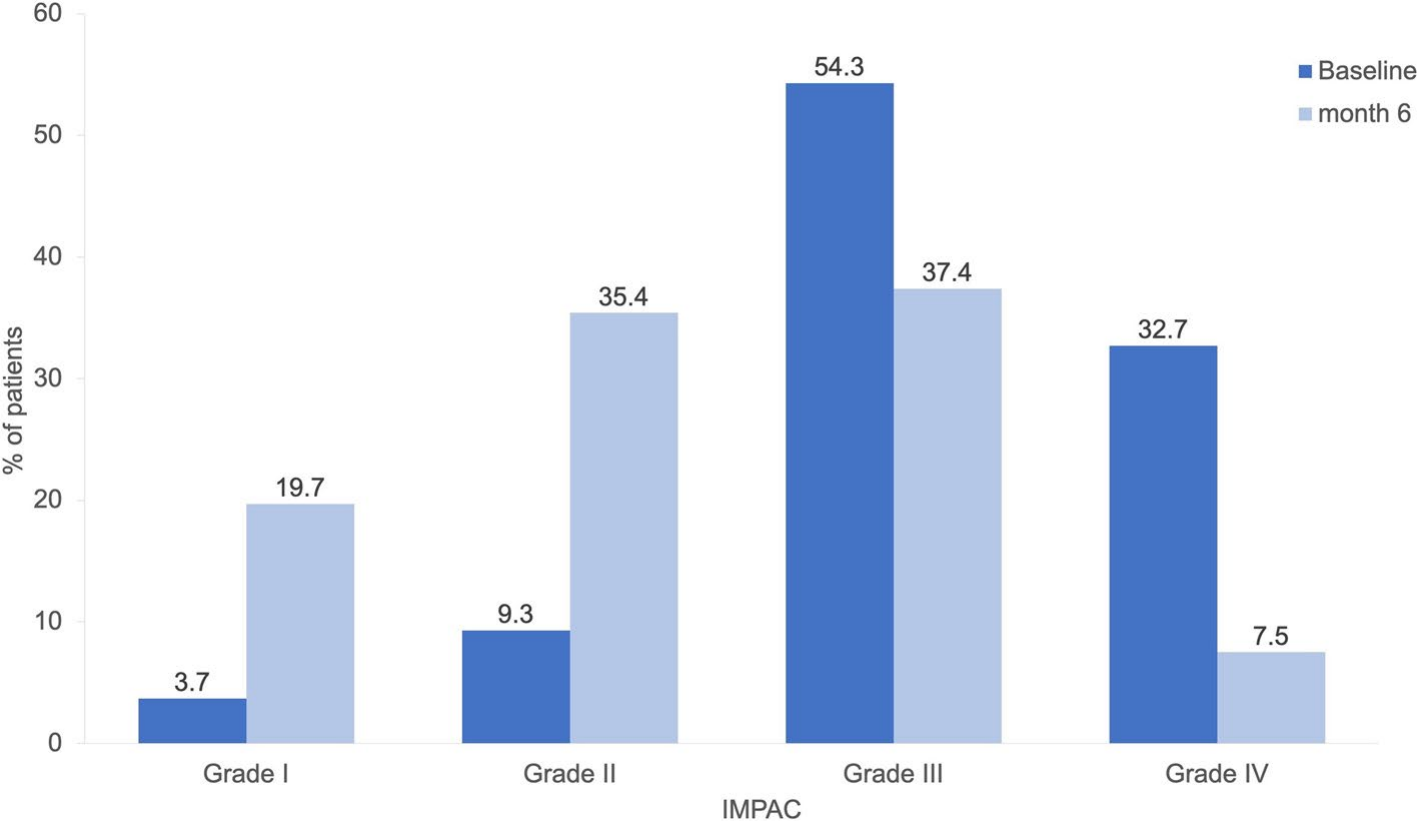
IMPAC grades at baseline (*N* = 162) and month 6 (*N* = 147)

The overall IMPAC score decreased by 6.1 ± 6.7 (*p* < 0.001) compared to baseline (12.6 ± 7.0) at month 6. There was an increase in percent of patients with grade I score from 3.7% at baseline to 19.7% at month 6. For Grade II, there was an increase from 9.3% at baseline to 35.4%. The percent of patients with grade III score reduced from 54.3% at baseline to 37.4%. Finally, for grade IV, the percent of patients decreased from 32.7% at baseline to 7.5% at 6 months (Fig. 4). Shifts towards the lower IMPAC grades over time were statistically significant at month 6 (*p* < 0.001).

#### Monthly Migraine Days (MMD)

The overall reduction in MMD was 7.6 ± 7.0 compared to baseline (*p* < 0.001). At baseline, MMD for patients with chronic migraine (CM) were 23.1 ± 5.3, decreasing to 12.5 ± 8.8 at month 6; a mean change of -10.7 ± 8.2 (*p* < 0.001). For patients with episodic migraine (EM), the baseline MMD were 11.0 ± 2.2, and were reduced to 5.9 ± 4.1 at 6 months; a mean change of -5.1 ± 4.4 (*p* < 0,001).

**Fig. 5:**
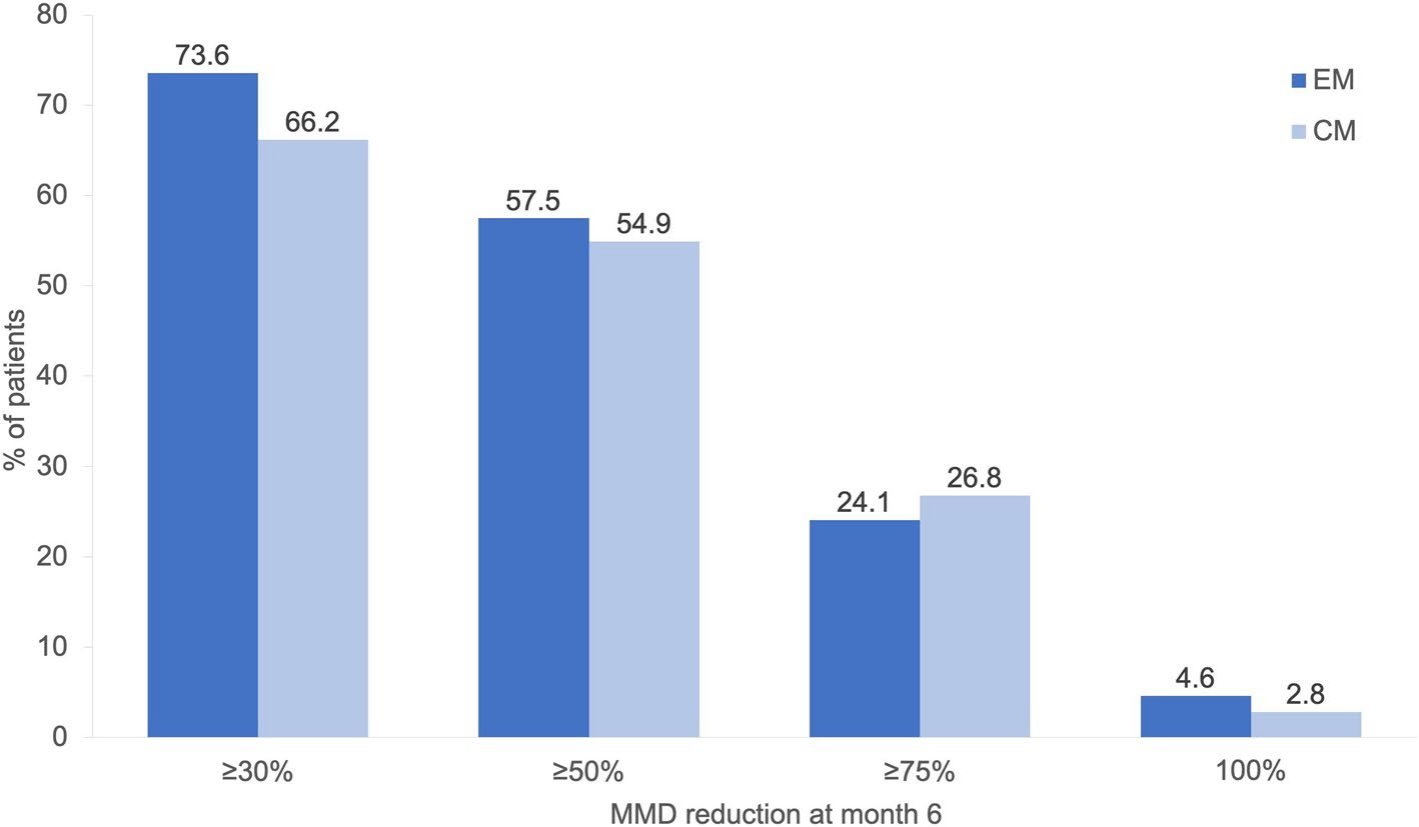
Response rates after 6 month per migraine type (*N* = 158). Abbreviations: MMD: monthly migraine days; EM: episodic migraine; CM: chronic migraine

Response rates were defined as percent of patients with an MMD reduction from baseline to follow-up of at least 30%, 50%, 75%, 100% of the baseline value. They are presented for EM and CM in Fig. 5.

#### Acute migraine-specific medication days

At baseline, patients had a mean number of migraine-specific medication days (triptan and/or ergot derivate) of 11.6 ± 7.0. At month 6, the mean intake was reduced to 6.6 ± 5.4 days (p < 0.001). The number of patients with medication overuse (i.e., 10 or more acute migraine-specific medication days [3]) decreased between baseline and 6 months, as shown in Table 3.

**Table 3:** Patients with ≥ 10 or < 10 acute migraine-specific medication days

Acute Migraine-Specific Medication Days	Baseline *N* = 145 (100%)	Month 6 *N* = 113 (100%)
≥ 10	81 (55.9%)	27 (23.9%)
< 10	64 (44.1%)	86 (76.1%)

#### Treatment satisfaction

Treatment satisfaction was measured by TSQM-9 (Treatment Satisfaction Questionnaire for Medication). At 6 months, a mean effectiveness of 67.1 ± 27.4, a mean convenience of 82.4 ± 16.2 and a mean global satisfaction of 72.4 ± 26.0 were reported. The TSQM-9 score was significantly higher in patients with EM than in patients with CM (*p* = 0.011) for effectiveness, but no such difference was observed for convenience (*p* = 0.065) or global satisfaction (*p* = 0.153). Treatment convenience and practicality were rated 7 and higher (out of 10) by 71% of patients at 6 months.

#### Dose adjustment of erenumab

All patients entered the study with 70 mg erenumab, administered once every month. After 6 months, a shift from 70 to 140 mg was seen in 39.5% of patients, with a higher rate of 140 mg in CM patients (*p* = 0.01).

### Safety

In total, 99 adverse events (AEs) were observed in 62 patients (Table 4). The most reported AE was constipation in 21.2%, followed by insufficient effect in 6.1%, lack of efficacy in 6.1%, diarrhea in 5.1% and injection site reaction in 2.0% of the study population. Also, 12 serious adverse events (SAEs) were observed in 11 patients. A total of 3/172 patients (1.7%) discontinued or interrupted erenumab in the first 6 months due to documented tolerability issues. An overview of all reasons for discontinuation are listed in the Supplementary Information (Table S2).

**Table 4:** Adverse and serious adverse events up to 6 months of observation

**Most reported adverse events (AEs)** n (%)	*N* = 99
Constipation	21 (21.2)
Insufficient effect	6 (6.1)
Lack of efficacy	6 (6.1)
Diarrhea	5 (5.1)
Injection site reaction	2 (2.0)
Serious adverse events (SAEs) n (%)	12 (12.1)
Anaphylactic shock^a^	1 (1.0)
Chest pain and arm pain left side	1 (1.0)
COVID-19	1 (1.0)
Inpatient headache therapy	2 (2.0)
Kidney stone	1 (1.0)
Medication overuse headache needing withdrawal in hospital setting	1 (1.0)
Meningioma	1 (1.0)
Mycoplasma pneumonia	1 (1.0)
Right motor and sensory syndrome and migraine	1 (1.0)
Transitory ischemic attack	1 (1.0)
Urinary tract infection	1 (1.0)

^a^Four months after erenumab discontinuation, hence no causal relationship assumed

*Abbreviations*: *AE* Adverse event, *SAE* Serious adverse event

## Discussion

Overall, this interim analysis of SQUARE confirms the key findings from the pivotal studies of erenumab in a real-world setting and adds new aspects to its growing body of evidence. Switzerland was the first country outside the US where the medication was approved.

To receive reimbursement of erenumab in Switzerland, patients must present with a minimum of 8 MMDs and must have made two or more unsuccessful treatment attempts with other prophylaxes. During the time of patient inclusion into this study, the mandatory starting dose for erenumab was 70 mg, with the possibility to increase to 140 mg at 3 months upon insufficient response (< 50%). Since March 2021, dosage can be chosen freely.

The interim results after the first 6 months were the aim of this analysis, with a main interest to observe the quality of life of patients suffering from episodic or chronic migraine. A special focus was on the social and family impact, which had not been investigated for erenumab or any other anti-CGRP pathway therapy so far.

In this interim analysis, a statistically significant decrease of migraine impact on quality of life from baseline to month 6 as assessed with HIT-6™ was observed in both episodic and chronic migraine, a finding comparable to those from phase III pivotal trials [12, 14]. These reductions in HIT-6™ are beyond the threshold of 3.7 points which is considered clinically meaningful [21]. The reductions in HIT-6™ scores found in this study after 6 months are numerically lower than those of a recent Italian real-world study [22] after the same time period. One explanation may be that a greater proportion of patients in the other study treated with 140 mg than in the present study.

The overall reduction in migraine-related disability, measured by mMIDAS, between baseline and month 6 was statistically significant. The mMIDAS decreased in both EM and CM, and the mMIDAS scores of CM patients approached those of EM patients after 6 months. A larger difference in the response between EM and CM was detected by mMIDAS. This finding can be explained by methodological restrictions of HIT-6™ – a relatively narrow range for potential changes – and some limitations by the background of both scales being originally developed for acute migraine therapy (triptans), where the impact of the individual attack is more relevant than frequency.

The IMPAC score, a measure for the impact of migraine on spouse or partner and adolescent children, showed a statistically significant decrease from baseline to month 6 in both EM and CM. This finding is of particular relevance as the effects of erenumab on this important dimension of the burden of migraine had not been evaluated to date.

An evaluation of the responder rates showed a ≥ 50% reduction of MMD in about 55% of patients in both migraine groups. The responder rates were relatively balanced between EM and CM patients after 6 months. Again, this is consistent with pivotal trials for erenumab and also with the results of a recent Italian real-world study [12–14, 22]. However, the responder rates for CM as well as the numerical reduction of MMDs in the real world were higher than expected.

Patient-reported satisfaction, measured by TSQM-9, was rated high with a mean score of 72.4 ± 26.0 out of 100 after 6 months. The “convenience” of the therapy was rated higher than “global satisfaction” and “effectiveness”. TSQM-9 scores were higher in EM than CM patients for the “effectiveness” domain, but not the “convenience” or “global satisfaction” domains. This result was expected since the administration of erenumab was the same for all patients. Although EM patients rated the “effectiveness” higher than CM patients, an impact on the “global satisfaction” could not be seen.

The adverse events documented in this interim analysis were consistent with already known events, and no unexpected serious or non-serious AE were submitted.

A limitation of this study is its single-arm design that was chosen due to low persistence of other prophylactic treatments in a real-world setting [10]. In addition, the patient population is relatively small compared to that of pivotal trials [12–14]. Finally, this being an interim analysis, not all data are fully mature and certain analyses will only be conducted following the completion of the study.

Taken together, patients treated with erenumab reported fewer migraine days per month, a lower impairment by their migraine symptoms and a reduction of the impact not only on themselves, but also on their social environment. These improvements across multiple measures are consistent with previous studies [22, 23]. All benefits were found for both EM and CM, across genders, and irrespective of PPTFs. As such, this study provides necessary evidence linking the findings from controlled clinical trials with erenumab to its application in standard clinical practice. This study shows that impact measures are as useful and important as the reductions in MMDs in the assessment of efficacy of migraine prevention treatments.

## Conclusions

In this observational study, erenumab has proven to be an effective and well-tolerated treatment for migraine prevention in adult patients with chronic and/or episodic migraine. The findings show that the therapeutic effects of erenumab observed in clinical trials are also evident in everyday clinical practice.

### Key findings

- After six months vs. baseline, erenumab treatment reduced Headache Impact Test (HIT-6™) scores by 7.7 ± 8.4 (primary endpoint) and monthly migraine days (MMD) by 7.6 ± 7.0, *p* < 0.001 for all.
- Compared with the overall treatment population, greater reductions in MMD were achieved in patients with CM (mean change of -10.7 ± 8.2) compared to patients with EM (mean change of -5.1 ± 4.4), *p* < 0.001 for all.
- Erenumab significantly reduced the Impact of Migraine on Partners and Adolescent Children (IMPAC) scores by 6.1 ± 6.7 (*p* < 0.001), thereby decreasing the impact of migraine on social and family life.

## Supplementary Information

**Additional file 1: Table S1.** Common observed PPTFs by medication. **Table S2.** Reason for erenumab dose discontinuation/interruption before month 6.

## Data Availability

The datasets used and/or analyzed during the current study are available from the corresponding author on reasonable request.

## Abbreviations

CGRP Receptor: Calcitonin gene-related peptide Receptor
AE: Adverse event
CM: Chronic migraine
eCRF: Electronic Case Report Form
EKNZ: Ethikkommission Nordwest- und Zentralschweiz
EM: Episodic migraine
HIT-6™: Headache Impact Test
ICHD-3: International Classification of Headache Disorders
IMPAC: Impact of Migraine on Partners and Adolescent Children
MMD: Monthly migraine days
mMIDAS: Modified Migraine Disability Assessment
MOH: Medication-overuse headache
PPTF: Prior prophylactic treatment failures
PRO: Patient-reported outcome
RAPS: Register of All Projects in Switzerland
SAE: Serious adverse event
SD: Standard Deviation
SQUARE: Swiss QUality of life and healthcare impact Assessment in a Real-world Erenumab treated migraine population
TSQM-9: Treatment Satisfaction Questionnaire for Medication
