# Correction: Swiss QUality of life and healthcare impact Assessment in a Real-world Erenumab treated migraine population (SQUARE study): interim results

**DOI:** 10.1186/s10194-022-01530-9

**Published:** 2022-12-19

**Authors:** Andreas R. Gantenbein, Reto Agosti, Christian P. Kamm, Gunther Landmann, Niklaus Meier, Gabriele Susanne Merki-Feld, Jens A. Petersen, Heiko Pohl, Philippe Ryvlin, Christoph J. Schankin, Dragana Viceic, Chiara Zecca, Elisabeth Schäfer, Ina Meyer, Michael E. Arzt

**Affiliations:** 1 Department of Neurology and Neurorehabilitation, ZURZACH Care, Bad Zurzach, Switzerland; 2 Kopfwehzentrum Hirslanden, Zurich, Switzerland; 3 Department of Neurology, Inselspital, University Hospital Bern and University of Bern, Bern, Switzerland; 4 Neurocentre, Luzerner Kantonsspital, Lucerne, Switzerland; 5 Center for Pain Medicine, Nottwil, Switzerland; 6 Department of Neurology, Spital Thun, Thun, Switzerland; 7 Department of Reproductive Endocrinology, University Hospital Zurich, Zurich, Switzerland; 8 Neurozentrum Bern, Bern, Switzerland; 9 Department of Neurology, University Hospital Zurich, Zurich, Switzerland; 10 Department of Clinical Neurosciences, CHUV, Lausanne, Switzerland; 11 Private Practice, Sion, Switzerland; 12 Neurocenter of Southern Switzerland, Ente Ospedaliero Cantonale (EOC), Lugano, Switzerland; 13 Faculty of Biomedical Sciences, Università Della Svizzera Italiana, Lugano, Switzerland; 14 Novartis Pharma Schweiz AG, Rotkreuz, Switzerland

**Correction: J Headache Pain 23, 142 (2022)**

**https://doi.org/10.1186/s10194-022-01515-8**

Following publication of the original article [1], the author group has identified an error in Fig. 1. The correct figure is given below.

**Fig. 1:**
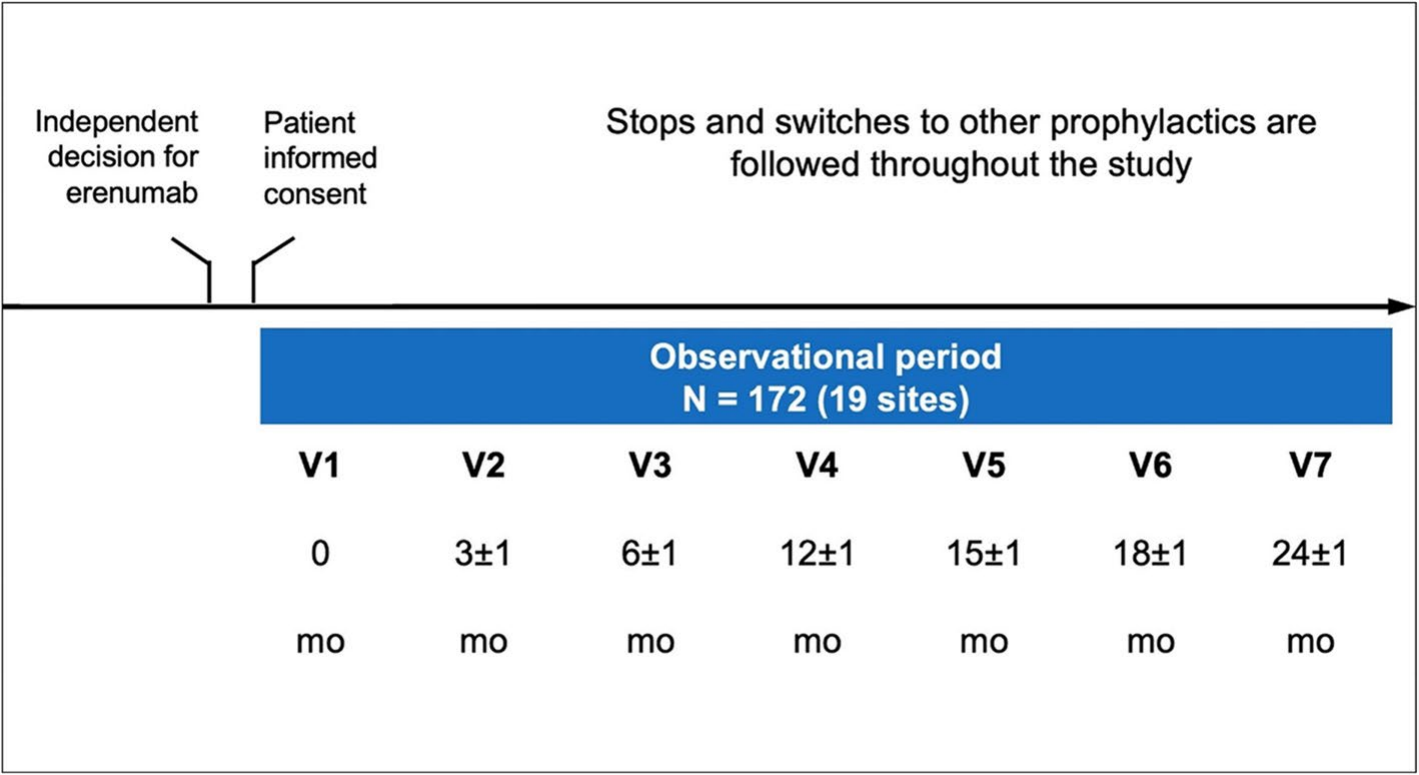
Study design and visit schedule. mo: month; V: visit

The original article [1] has been corrected.
