# Supplementary material for: Swiss QUality of life and healthcare impact Assessment in a Real-world Erenumab treated migraine population (SQUARE study): interim results
Source: J Headache Pain. 2022 Nov 18;23(1):142. doi: 10.1186/s10194-022-01515-8 (PMC9673191; doi:10.1186/s10194-022-01515-8)
Supplement: Supplementary file 1 — Additional file 1: Table S1. Common observed PPTFs by medication. Table S2. Reason for erenumab dose discontinuation/interruption before month 6. [file 10194_2022_1515_MOESM1_ESM.docx]

**Table S1. Common observed PPTFs by medication**

| **PPTF - Nature** n (%) | N=172 |
| --- | --- |
| Propanolol/ Metoprolol | 138 (80.2) |
| Other betablockers or antihypertensives | 32 (18.6) |
| Topiramate | 134 (77.9) |
| Other antiepileptics | 33 (19.2) |
| Flunarizine | 58 (33.7) |
| Botulinum toxine | 27 (15.7) |
| Antidepressants | 86 (50.0) |
| Nutritional supplements | 92 (53.5) |
| Devices | 48 (27.9) |
| Other | 33 (19.2) |

Abbreviations: PPTF: Prior Prophylactic Treatment Failure

**Table S2.** Reason for erenumab dose discontinuation/interruption before month 6.

| Reason for discontinuation n (%) | 18 (100) |
| --- | --- |
| Insufficient effect | 3 (16.7) |
| Lack of efficacy | 3 (16.7) |
| Monetary / reimbursement | 3 (16.7) |
| Unknown | 1 (5.6) |
| Lost to follow up due to migraine attack | 1 (5.6) |
| Poor tolerability | 3 (16.7) |
| Desire for children | 1 (5.6) |
| Withdrawal of ICF | 3 (16.7) |

Abbreviations: ICF: informed consent form.
